# Westminster Hospital Students' Dinner

**Published:** 1893-11-25

**Authors:** 


### WESTMINSTER HOSPITAL STUDENTS' DINNER.

The above event took place at the Criterion Restaurant last Thursday. Dr. Bourns presided. The affair was in every way a success.

Mr. De Renzi, in an able speech, remarked on the various changes in the staff since the last annual dinner, and special reference was made to the retirement of Dr. Sturges from the lectureship in medicine, and also to Mr. Davy, who has lately resigned his position as surgeon to the hospital. Mr. De Renzi said that the students all owed a great debt of gratitude to the members of the staff, who took such an interest in the students individually and collectively, and on behalf of his colleagues and himself warmly thanked the staff for their unvarying kindness and courtesy. He warmly welcomed Dr. Wills and Dr. Gorsage as the newly-elected members of the staff, and said that he felt sure it was a source of gratification to all that they were both Westminster men, and that no doubt existed as to the reputation of Westminster as a hospital and medical school being worthily upheld. With the hospital itself few could complain; it was abundantly supplied with material for clinical teaching. The medical school showed signs of increasing prosperity, as evidenced by the larger number of entries this year as compared with the years immediately preceeding. Some critical remarks were then made with regard to the system of antiseptics as carried out at the hospital, and in connexion with this subject Mr. De Renzi said he thought it was a fitting opportunity for those present, as representing a metropolitan hospital, to pay a tribute of respect to Sir Joseph Lister, whose name would be handed down to posterity as the greatest benefactor of our time. Allusion was made to the nursing of the hospital, and it was suggested that a more frequent change of probationers in the wards might be beneficial. The list of toasts was a large one, and the dinner wes followed by a concert, to which Messrs. Mills, Twemlow, Nitch-Smith, Evans, Fleming, White, Baxter, and Towne contributed.

